# Study protocol: national research partnership to improve primary health care performance and outcomes for Indigenous peoples

**DOI:** 10.1186/1472-6963-10-129

**Published:** 2010-05-19

**Authors:** Ross Bailie, Damin Si, Cindy Shannon, James Semmens, Kevin Rowley, David J Scrimgeour, Tricia Nagel, Ian Anderson, Christine Connors, Tarun Weeramanthri, Sandra Thompson, Robyn McDermott, Hugh Burke, Elizabeth Moore, Dallas Leon, Richard Weston, Haylene Grogan, Andrew Stanley, Karen Gardner

**Affiliations:** 1 Menzies School of Health Research, Institute of Advanced Studies, Charles Darwin University, Darwin, Australia; 2 Centre for Chronic Disease, School of Medicine, University of Queensland, Brisbane, Australia; 3 Centre for Indigenous Health, University of Queensland, Brisbane, Australia; 4 Centre for Population Health Research, Curtin Health Innovation Research Institute (CHIRI), Curtin University of Technology, Perth, Australia; 5 Centre for Health and Society, School of Population Health, University of Melbourne, Melbourne, Australia; 6 Discipline of Public Health, University of Adelaide, Adelaide, Australia; 7 Aboriginal Health Council of South Australia, Adelaide, Australia; 8 Northern Territory Department of Health and Families, Darwin, Australia; 9 Western Australia Department of Health, Perth, Australia; 10 Centre for International Health, Curtin University of Technology, Perth, Australia; 11 Combined Universities Centre for Rural Health, University of Western Australia, Geraldton, Australia; 12 Division of Health Sciences, University of South Australia, Adelaide, Australia; 13 Maari Ma Health Aboriginal Corporation, Broken Hill, Australia; 14 Aboriginal Medical Services Alliance Northern Territory, Darwin, Australia; 15 Queensland Aboriginal and Torres Strait Islander Health Council, Brisbane, Australia; 16 Aboriginal and Torres Strait Islander Community Health Service - Brisbane Ltd, Brisbane, Australia; 17 Department of Health, Queensland Government, Brisbane, Australia; 18 Department of Health, South Australian Government, Adelaide, Australia; 19 Australian Primary Health Care Research Institute, Australian National University, Canberra, Australia

## Abstract

**Background:**

Strengthening primary health care is critical to reducing health inequity between Indigenous and non-Indigenous Australians. The Audit and Best practice for Chronic Disease Extension (ABCDE) project has facilitated the implementation of modern Continuous Quality Improvement (CQI) approaches in Indigenous community health care centres across Australia. The project demonstrated improvements in health centre systems, delivery of primary care services and in patient intermediate outcomes. It has also highlighted substantial variation in quality of care. Through a partnership between academic researchers, service providers and policy makers, we are now implementing a study which aims to 1) explore the factors associated with variation in clinical performance; 2) examine specific strategies that have been effective in improving primary care clinical performance; and 3) work with health service staff, management and policy makers to enhance the effective implementation of successful strategies.

**Methods/Design:**

The study will be conducted in Indigenous community health centres from at least six States/Territories (Northern Territory, Western Australia, New South Wales, South Australia, Queensland and Victoria) over a five year period. A research hub will be established in each region to support collection and reporting of quantitative and qualitative clinical and health centre system performance data, to investigate factors affecting variation in quality of care and to facilitate effective translation of research evidence into policy and practice. The project is supported by a web-based information system, providing automated analysis and reporting of clinical care performance to health centre staff and management.

**Discussion:**

By linking researchers directly to users of research (service providers, managers and policy makers), the partnership is well placed to generate new knowledge on effective strategies for improving the quality of primary health care and fostering effective and efficient exchange and use of data and information among service providers and policy makers to achieve evidence-based resource allocation, service planning, system development, and improvements of service delivery and Indigenous health outcomes.

## Background

### Indigenous health and primary health care

The picture of Indigenous health disadvantage is well reflected in the recent study of Indigenous burden of disease: "The health gap in diseases and injuries between Australian Indigenous and general populations is unacceptably large. At every age, young or old, Indigenous Australians are sicker, and die earlier, than their non-Indigenous counterparts" [1].

Primary Health Care is defined as "socially appropriate, universally accessible, scientifically sound first level care provided by a suitably trained workforce supported by integrated referral systems and in a way that gives priority to those most in need, maximises community and individual self-reliance and participation and involves collaboration with other sectors. It includes health promotion, illness prevention, care of the sick, advocacy and community development [2]." International evidence has demonstrated that stronger primary care systems are associated with reduced premature mortality [3]. Enabling primary health care services to respond more effectively to the ongoing demands of providing acute care as well as the range of functions described in the above definition, including specifically the increasing demands of chronic illness care, is a major challenge for policy makers, managers and practitioners.

### Lack of national data on quality of primary health care

The quality of diabetes care has been relatively widely studied and provides some insights into the quality of primary health care. However, our recent search of the websites of national level health departments of five countries (Australia, New Zealand, USA, Canada and the United Kingdom) for publicly released data on diabetes care reveals that Australia has poorly developed systems to report on quality of diabetes care at the primary care level, both for general and Indigenous populations [4]. In contrast, New Zealand and the UK have implemented routine systems to monitor diabetes care in primary care settings [5,6]. New Zealand also has designated systems to monitor diabetes care among its indigenous people.

Data on other major conditions which are managed largely in primary care services are perhaps even more deficient than for diabetes. For hypertension (estimated to affect about 14% of Australian adults [7]), and hypercholesterolaemia (estimated to affect 50% of people aged 25-64 years [8]) the AIHW reports that there is little information on how these conditions are managed in primary health care [9]. For renal disease the regular data collected and reported are only for people with End Stage Renal Disease who receive dialysis or kidney replacement therapy [9].

While there is a lack of national data regarding diabetes primary care among Indigenous Australians, several studies conducted in the Northern Territory and Queensland reported that most Indigenous people with diabetes did not achieve adequate glycaemic control [10-12]. For example, of Indigenous patients with HbA1c tested in the previous year, less than one-third had their HbA1c less than 7.0%. This considerable gap between recommended diabetes care and care patients actually receive shows that achievable benefits are not being delivered by our healthcare systems.

### Improving primary health care performance and data through quality improvement initiatives

Modern Continuous Quality Improvement (CQI) aims to facilitate ongoing improvement by using objective information to analyse and improve systems, processes and outcomes [13,14]. Key features of modern CQI approaches make them well suited to the Indigenous Australian setting. The participatory approach and "customer focus" of CQI, and the combination of scientific and humanistic values [15-17],. fits with the requirement to take account of the principles and values of Aboriginal and Torres Strait Islander people, as expressed in recent national statements on research and cultural respect [18,19].

There is strong 'grass roots' interest in clinical CQI among Aboriginal Community Controlled Health Services (ACCHSs). This interest in CQI has been fostered and supported by a range of funding and program initiatives, both within the community controlled sector and government health departments. These include: increasing uptake of computerised clinical information systems; wider implementation of accreditation; introduction of Key Performance Indicators; and funding streams such as the Healthy for Life Program. The interest from the Indigenous primary health care sector is amply demonstrated by the voluntary uptake by services of the Continuous Improvement Projects, the Healthy for Life Program, the National Primary Care Collaboratives and the Audit and Best Practice for Chronic Disease Project [20].

### The Audit and Best Practice for Chronic Disease (ABCD) Project

Informed by modern CQI theory and practice, the ABCD Project commenced in 2002 as a quality improvement initiative designed to support Indigenous services to assess and improve their systems for the delivery of best practice care. The initial focus of the project was on the prevention and management of chronic disease in 12 Aboriginal community health centres in the Top End of Northern Territory (NT) (2002-2005). The project subsequently broadened its scope to include maternal and child health care, and has developed prototype audit tools for primary mental health care and for prevention and management of rheumatic heart disease. Work on developing tools to support quality improvement in health promotion, food supply and the community environment is underway. By the end of 2009 the ABCD Extension Project was supporting the participation of over 60 Aboriginal community health centres from 4 states/territories (2005-2009) and the tools had been used by at least another 60 primary health care services.

**Figure 1:**
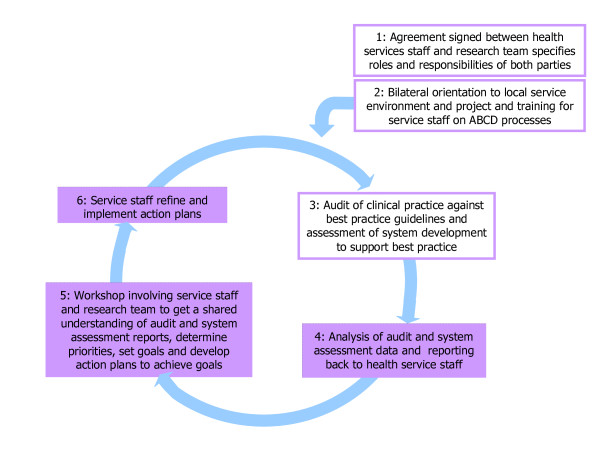
**Outline of the ABCD approach**.

The ABCD Project featured annual cycles of system assessment and audits of clinical records to assess the quality of care, feedback workshops, goal setting and action planning, and implementation of system changes (see Figure 1). The facilitated quality improvement (QI) cycle requires engagement of health services staff in assessment, interpretation of data, priority setting, planning and implementation [21]. A hub coordinator was located in each region to support and facilitate the execution of QI cycles.

In the original ABCD Project all 12 participating services achieved significant improvements in systems development, processes of diabetes care and patient outcomes.

Interim analysis of data from the ABCD Extension Project in 2008 showed that 42 of the 62 participating services had completed at least two rounds of data collection. Improvements observed included: increase in the proportion of diabetes patients with ideal HbA1c control from 24% to 35%; and increase in the proportion of evidence-based preventive services delivered to healthy adults from 32% to 42%. A final report on the ABCD Extension Project will be produced in early 2010.

### Understanding variation in and determinants of quality of care

**Figure 2:**
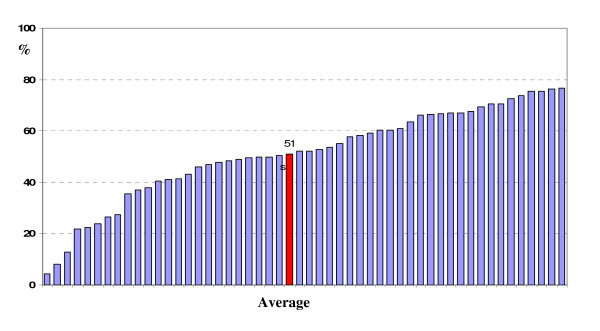
**Overall delivery of diabetes services by health centre (includes data for participating service up to 2008)**. The overall adherence to delivery of scheduled services for each patient was calculated by dividing the sum of services delivered by the total number of scheduled services and expressing this as a percentage. Each bar in the chart represents the average for overall delivery of scheduled services for clients in each health centre participating in the project. The highlighted bar shows the average for all health centres.

Our work to date provides insight into quality of care and its variation within and between Indigenous primary health care settings. Baseline data show wide variation in quality of diabetes care across health centres (Figure 2). For example, overall 51% of diabetes services specified by evidence-based guidelines were delivered to patients, with large variation between health centres (range 4%-77%). Similarly, the wide variation in quality of primary care is evident across a range of other indicators. For example: A) Preventive services to well adults: overall 32% of preventive services recommended by guidelines were delivered to well adults, with a range of 2%-74% between health centres; B) Maternal health care: for women who had given birth in the past year the proportion whose first antenatal visit was in the first 12 weeks of gestation ranged from 20% to 70%; and C) Child health care: for children under five years of age the proportion with a record of delivery of clinical services such as weight checks, ear examinations, hearing checks and developmental assessments in line with age specific guidelines ranged from 26% to 78% [22].

Previous studies have investigated state, health care facility, clinician and individual client level characteristics as determinants of quality in primary health care [23-27]. Multilevel statistical modelling studies [24,26]. indicate that individual client level characteristics are responsible for a large proportion of variation in quality of care, with less variation explained by clinician and facility factors. Studies of team culture and climate and nurse-doctor composition of the clinical team have shown no or limited association with quality of care [28,29]. Observational studies suggest larger clinical teams and practices which are more likely to respond to financial incentives deliver higher quality clinical care [30]. The literature shows a clear need to better identify what factors contribute to performance difference at the facility level, including characteristics of patient population and the facility. Understanding the causes of variability is a key to developing and implementing targeted quality improvement programs. Understanding and managing variation should assist policy makers, managers and clinicians to align the capacity of health care systems and organisational processes with desired results.

### Translation of ABCD research findings into policy and practice

**Figure 3:**
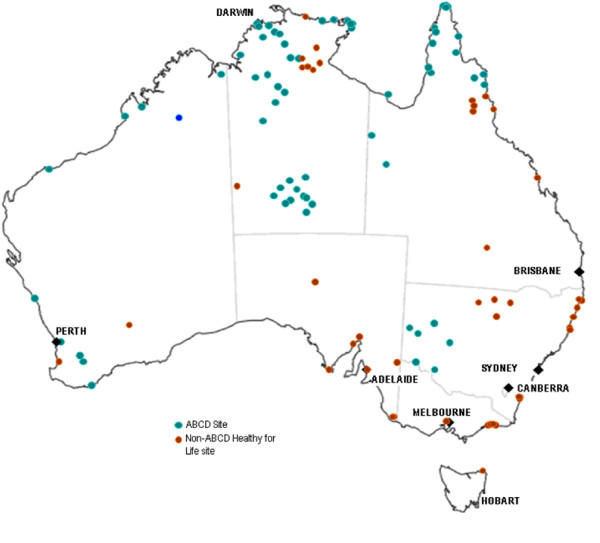
**ABCD sites and associated Healthy for Life sites using the ABCD tools (December 2009)**.

Findings and knowledge gained from the ABCD project have been translated into the policy implementation process and clinical practice, including: 1) the development and implementation of the Federal Government's Healthy for Life program. System assessment and clinical audit tools used in the study have been included in the Healthy for Life toolkit. By December 2009 there were over 100 health centres (including 62 ABCD sites, see Figure 3) across all states/territories using these tools; 2) the NT Department of Health and Families has adopted the ABCD CQI process as routine practice to be implemented across all government funded centres, with creation of regional-based CQI coordinator positions to support its implementation; 3) the Queensland Health Department is implementing the ABCD process across North Qld and exploring potential implementation in central and southern Qld; 4) Maari Ma Aboriginal Health Corporation in Far West NSW has been using the ABCD process to support and evaluate implementation of their Chronic Disease Strategy over the past four years; and 5) four of the five ABCD hub coordinators were employed by state/territory health departments or Aboriginal Community Controlled Health Organisations, reflecting a strong commitment of health authorities to support implementation of the project processes.

### Challenges faced by the ABCD Project and opportunities for accelerating improvements

Research funding for the project came to an end in December 2009. With the wide engagement of service organisations we aim to establish mechanisms to a) provide ongoing support for services in implementing CQI and b) to continue our program of research on primary care quality improvement. The geographic dispersion of participating services poses logistic and management challenges for the research team to support hub coordinators and health services at the regional level. There is an increased need to provide integrated management, clinical and research support to hub coordinators who are working with a diversity of services in various local circumstances. The commitment by health authorities provides potential to address some of these requirements. The NHMRC Partnership initiative now offers an ideal funding mechanism to build on the national ABCD research network. This will in turn inform ongoing efforts in development of effective mechanisms to provide routine support to services wishing to engage in QI processes.

By building on a national Indigenous primary care quality improvement network which links researchers directly to users of research (service providers, managers and policy makers), our partnership will foster effective and efficient exchange and use of data and information among service providers and policy makers to achieve evidence-based resource allocation, service planning and system development and innovation. This new research partnership has important potential to address local needs and achieve local improvements while simultaneously creating knowledge that can be applied more broadly.

### The Aim and objectives

The partnership will focus on improving the quality of Indigenous primary health care (specifically prevention and management of chronic disease and maternal and child health care) through:

1) investigating the variation in quality of care between primary health care centres and between regions;

2) exploring the factors associated with clinical performance of primary health care centres at the health centre and regional level;

3) identifying and examining specific strategies that have been effective in improving primary care clinical performance; and

4) working with health service staff, management and policy makers to enhance the effective implementation of successful strategies.

A major complementary aim of the Partnership will be to ensure the effective translation of research findings arising from the Partnership into clinical practice and policy. The Partnership will strengthen and support the integrated approach to research translation that has been shown to be effective in the current national network of the ABCD Project, specifically in building capacity and providing a strong institutional base for engagement between researchers, clinicians, health service staff, managers and policy makers in identification of priority research issues within the scope of the Partnership, supporting development and implementation of research into these issues, participating in interpretation of data, and development of strategies to achieve improvements in care and health outcomes.

## Methods/Design

### Partnership arrangements to support CQI cycles and research transfer

We plan to continue to use the governance model that we have used successfully for the ABCD Project to date. The established and proven governance and management framework provides mechanisms for productive partnerships with government and community-controlled organisations, timely communication between partners, and effective translation of research evidence into policy and practice.

**Figure 4:**
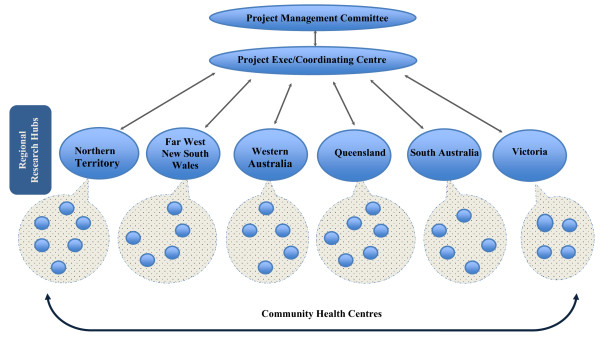
**Project management structure**.

As illustrated in Figure 4 and Table 1, the partnership project will be managed by a Management Committee made up of the nominated Chief Investigators, the nominated contact person for each industry partner and the Partnership Project Coordinator. The investigators have been specifically selected for their research expertise, understanding of Indigenous primary health care, connections with the Indigenous community controlled primary health care sector; and understanding of and involvement in Indigenous primary health care policy and management processes at regional and national levels. We are interested in extending the project through inclusion of partners in other jurisdictions.

**Table 1:** Research and Policy/Service Partners

Regional Research Hub	Supporting Research Institution	Policy/Service partner
**Northern Territory** **(NT)**	Menzies School of Health Research	Northern Territory Department of Health and Families
		Aboriginal Medical Service Alliance of the Northern Territory (AMSANT)
**Far West New South Wales (FW NSW)**	Menzies School of Health Research/University of South Australia	Maari Ma Aboriginal Health Corporation
**Western Australia (WA)**	Curtin University of Technology	Department of Health of Western Australia
**Queensland (QLD)**	University of Queensland	Queensland Department of Health
		Queensland Aboriginal and Islander Health Council (QAIHC)
**South Australia (SA)**	University of South Australia	Aboriginal Health Council of South Australia (AHCSA)
		South Australian Department of Health
**Victoria (Vic)**	University of Melbourne	under negotiation

At the national project level, activities will be conducted and supported by a project coordinating centre comprising a small project executive team and core staff with skills in project management and administration, data management and analysis and reviewing research literature.

The lead chief investigator in each region will be responsible for establishing a Regional Research Hub, with support from a Regional Hub Steering Committee comprising chief investigators and associated investigators in each region, the nominated contact person for each industry partner in that region and other key individuals as identified at a regional level. Funding contributions from partner organisations in each region will be used to support the work of a researcher in that region.

As an operational base in each region, the Regional Research Hub will 1) support participating health centres in their region in the implementation of successive CQI cycles; 2) provide a channel for support from the project coordinating centre to participating health centres and for reporting back to the project coordinating centre; and 3) foster and strengthen partnerships between researchers, health service managers/providers and policy makers at the regional level, in order to proactively translate research evidence into health policy and practice.

The Partnership Project will be supported by a web-based information system at http://www.one21seventy.org.au. The automated analysis and reporting function of the website provides for immediate access by health centre staff and management of a wide range of performance indicators, including trends over time and comparisons with other de-identified services. Reports are also generated as MS Word documents which allows for editing and use of these reports for a wide range of requirements by health centres and regional health authorities. The website also provides access for services to a range of Project and other related resources. The web-based database allows for download and analysis of data for research purposes where there has been formal agreement by health centre management to participate in the research and ethics approval has been obtained. This requirement is in separate to and in addition to any agreement regarding use of the One21seventy website for quality improvement purposes.

The Partnership will strengthen and support the integrated approach that has been shown to be effectively operating in the ABCD Project. Key aspects of this approach include:

1. the conceptualisation and ongoing refinement of the ABCD CQI approach in line with international research evidence on achieving improvement in clinical practice, diffusion of innovations, and Indigenous research values, ethics and research priorities and to specifically apply this evidence to informing a program of research aimed at improving Indigenous health;

2. the wide implementation of a standard set of tools that are designed to generate performance indicators which reflect adherence to evidence based clinical practice guidelines across a large number of Indigenous primary health care services nationally;

3. support for the implementation of these tools with detailed protocols, specific training and experienced regional quality improvement coordinators;

4. the engagement of working groups with specific clinical expertise and understanding of evidence based clinical guidelines to support regular updating and refinement of clinical audit tools;

5. the involvement of service staff in use of these audit tools to audit health centre clinical records, thus increasing and maintaining familiarity with current best practice guidelines and raising awareness of deficiencies in clinical record systems and of discrepancies between best practice guidelines and practice as documented in clinical records;

6. the use and ongoing refinement of a tool to assess systems to support clinical best practice, for the purpose of engaging health centre staff in efforts to improve health centre systems drawing on current research evidence on effective systems;

7. the use of a web-based system for data entry and automated analysis and reporting to ensure locally relevant data are available in a meaningful format to health centre staff and regional health authorities within a short time of completing the clinical audits and systems assessments;

8. the use of a network of regional research hubs, skilled facilitators and clinical experts to support health centre staff in interpreting their data, identifying priorities for action and developing action plans to achieve improvement, with an emphasis on the opportunities for translation of research evidence in each of the above steps;

9. engagement of a wide network of clinical staff, health service managers, policy makers and researchers in an integrated quality improvement initiative which draws on relevant clinical audit data and health centre systems data, clinical, management and policy experience and expertise and relevant research evidence; and

10. production of publications in peer reviewed scientific journals; production and dissemination of policy briefs and fact sheets targeting bureaucrats, politicians and health service staff and management; and presentation of conference papers to support wider research translation beyond the Partnership Project network.

### Quality improvement tools and data collection

1. Health centre system assessment: a System Assessment Tool (SAT) developed in our ABCD study (through modification of the Chronic Care Model and its associated Assessment of Chronic Illness Care scale [31]) will continue to be refined and used to evaluate the state of community health centre system development with regard to prevention and management of chronic illness and maternal and child health care. The SAT provides a broad assessment of key components of health centre systems which have been identified through international research as being important to supporting best practice chronic illness care [32-34]. The SAT includes items that are grouped into five components (delivery system design, self-management support, decision support and clinical information systems, external linkages, and organisational influence and integration). Based on health centre staff consensus, each item is given a score indicating the state of development, ranging from 0 (not at all) to 11 (fully developed). Health staff will be asked to provide qualitative justification (e.g. description of facilities/activities) for their scoring. As part of the health centre system assessment, community and health centre contextual information will also be collected using a structured supplementary questionnaire.

The mean is calculated from individual item scores to create a component score, and the mean of the component scores forms the overall system score for the community health centre. The SAT serves both as a measurement tool and developmental tool, as the discussion of system components leads to better understanding among staff of the quality of systems and consideration of how systems could be improved [35].

2. Qualitative research methods: Subject to the priorities of the research partners in each region, quantitative and qualitative data provided by the clinical audits and the SAT will be used to guide further qualitative research into health centre level or regional level factors associated with variation in clinical performance. The general approach will be to include 4 to 6 health centres in each region based on the response to an invitation for expressions of interest in being involved in this qualitative component of the work. The sample might include Indigenous health workers and other staff in these health centres and a random sample of health centre clients, subject to the specific aims of the research and the approval of an appropriately constituted research ethics committee. The client sample might include infrequent and regular attenders, with the intention of getting a diversity of views. Qualitative data may be obtained through interviews or focus groups at regional and local health centre levels.

This qualitative component of the work is planned to occur in three phases. Phase 1 (six months): recruitment of regional researchers; engagement of experienced qualitative researchers; methods and training workshop. Phase 2 (12 months): consultation and engagement of services; conduct of interviews, local feedback and action. Phase 3 (six months): analysis workshop at national project level; write-up, refinement of tools and process; feedback and training at regional levels.

3. Clinical record audits: We have developed, used and refined a number of audit tools and protocols (including detailed sampling processes) over several years. The use and findings from the application of these tools have been described in a number of publications [22,36,37]. The tools to be used in this Partnership Project include: i) a vascular and metabolic syndrome clinical audit tool. This covers diabetes, CVD, hypertension and renal disease management. ii) a preventive service clinical audit tool; iii) a maternal health clinical audit tool; and iv) a child health audit tool. We aim to have a sample of at least 30 client records to be audited at each centre for each of the health conditions which individual centres choose to focus on. If there are more than 30 eligible clients in a health centre, a random sample of 30 or more records may be drawn. In the centre where there are fewer than 30 eligible clients, records of all clients are included. Trained data abstractors will conduct clinical record audits at participating health centres. Our previous studies showed a Kappa statistic for intra-rater reliability of between 0.74-1.00 for an audit of diabetes care ^39 ^and between 0.79 and 0.93 for an audit of preventive care [37].

The above tools form the assessment step of the integrated quality improvement cycle (Figure 1) that will be implemented in participating health centres with the support of regional staff. Access to the tools and website at a regional and local level will be supported through funding agreements between relevant health authorities and One21seventy (the National Centre for Quality Improvement in Indigenous Primary Health Care) or in special circumstances through specific research funding agreements.

Researchers in each region will have a role with health services in supporting the integrity of the QI cycle and enhancing data quality through participating in training, monitoring and feedback. The project researchers will also have a key role in analysis and interpretation of audit and system assessment data.

### Outcome measures

Outcome measures will be based on data generated by use of the quality improvement tools and from quarterly hub reports and will be defined in terms of:

1. Intermediate health outcomes (e.g. control of HbA1c, control of blood pressure, birth outcomes, child growth, and prevalence of childhood diseases). The relationship of intermediate health outcomes (such as HbA1c control and BP control) to more definitive outcomes (such as the development of complications) has been well demonstrated by international research. Data on these outcomes are well documented in clinical records and we have used these data in a number of studies to date [22,36-38].

2. Clinical service performance (proportion of clients for whom services specified in the clinical practice guidelines are delivered, mean proportion of guideline specified services delivered for all clients). The audits of clinical service performance focus on services for which there is the most substantial evidence base for effectiveness. On the strength of the evidence base behind the clinical guidelines the effective delivery of these services is expected to impact on health outcomes. We have used audits of delivery of these services in a number of studies to date [22,36-38].

3. Improvements in the quality of organisational systems as reflected by the SAT scores and scope and depth of system changes initiated and implemented by health centre staff.

### Data analysis in relation to Partnership aims

#### Aim 1)

Investigating the variation in quality of care between primary health care centres and between regions. Quantitative data arising from the clinical audit tools will be analysed to describe the variation in care according to best practice guidelines.

#### Aim 2)

Exploring the factors associated with clinical performance of primary health care centres at individual patient level and systems level.

The quantitative analysis to examine factors associated with variations in care uses multilevel random effects regression models (linear or logistic). Our data have inherent multilevel, dependency structure, as quality of care data collected at the individual patient level are clustered within health centres which in turn are clustered within jurisdictions. A range of factors measured using the community and health service survey and the systems assessment tool will be included in the regression models. This will allow us to assess, for example, associations between health care organisation factors and quality of care, with adjustment of patient, community and contextual factors. We can also quantify to what extent those associations are modified by policy factors over time or whether factors at the organisational level such as leadership and team work mediate the affects of poor workplace on improvement in performance. This partnership project will recruit a minimum of 60 health centres with a diabetes audit sample of approximately 1500 (based on the ABCD data). Taking into account the dependency structure of our data (with a design effect of 1.84), a sample size of 1500 should yield a power of 95% in testing associations between health care organisation factors and overall delivery of diabetes care, at a 0.05 significance level http://www.ats.ucla.edu/stat/stata/dae/powerreg.htm.

The approach to analysis and reporting on the qualitative data obtained at regional and local health centre levels will include grouping of data according to themes, comparison and contrasting within and between groups of interviewees, triangulation of findings against systems assessment findings, feedback, reflection and action planning with health centre teams, and clear documentation of process and findings at each stage in each region. At the national project level we will conduct a workshop to compare and contrast process, findings and actions between regions, and conduct an overall synthesis of qualitative findings.

#### Aim 3)

Examining specific strategies that have been effective in improving primary care clinical performance.

We will undertake time series analyses to examine the impact of the QI initiatives. Time series analysis has been specified by the Cochrane Effective Practice and Organisation of Care Group as feasible and appropriate to assess effectiveness of an organisational change intervention (such as the QI process proposed in our application) on health care.

We will assess the impact of the QI strategies by categorising sites according to the depth and integrity of the implementation of the intervention as reflected in system assessment data and related qualitative data. Dose-response relationship will be used to assess strength of evidence of causal relationship between implementation of QI strategies through the partnership and improvement in quality of care. This is an approach that we piloted in the original ABCD Project, and which we are developing further and are applying to the analysis of the current ABCD Project data. In this approach sites where there has been poor implementation or where the integrity of the QI process has been disrupted effectively act as controls, with statistical adjustment of potential confounders (including co-existence of initiatives/programs other than our intervention) as measured through a community and health service survey and the systems assessment tool.

#### Aim 4)

Working with health service staff, management and policy makers to enhance the effective implementation of successful strategies. Consistent with the action research approach used in the ABCD Project, the findings of the Partnership Project will be used to influence practice, systems and policy throughout the duration of the project. The regular meetings of the project executive and of the project management committees will be used by the research and industry partners to ensure the research program addresses service and policy priorities and to discuss emerging findings and their implications for policy and service delivery. This approach is designed to facilitate policy and service sector involvement in development of the research and to enable early formulation of management and policy responses to findings. The ongoing collection, analysis and interpretation of data will allow us to examine the impact of management and policy responses, and to consider how responses may be refined over time.

### Ethics approval

The main research ethics issues for the Project are 1) the auditing of client health records held in health centres without the consent of the clients; 2) the protection of the privacy and the confidentiality of client records, qualitative interview data, and of data related to specific health centres; 3) appropriate use of project data for the benefit of participating services and the wider community. We have developed strict privacy and confidentiality processes and procedures for protecting the privacy of clients and the identity of participating services in the reporting of project findings. In our submissions to the research ethics committees in all current participating regions for the ABCD Project the ethics committees have accepted that obtaining individual client consent to audit health records would render the project impractical; that the benefits of the project far outweigh the small risks to privacy of client records; and that the sort of audit methods used in the project are used in many services as a standard approach to quality improvement and quality assurance. The web based information system will automatically generate reports for each health centre. These reports will be accessible via the website only to health centre staff and specified members of the research team. The research team will not make these reports available to any other party without the written agreement of health centre management. The Health Centre's data will be included in a data pool that will allow anonymous regional comparisons for each variable to be included in the Health Centre's report. The data will be further analysed for research purposes by the research team under strict conditions of confidentiality of research data.

We have obtained the ethics approval from the Human Research Ethics Committee of NT Department of Health and Families and Menzies School of Health Research (including its Indigenous Health Research Ethics Committee) (Reference number 09/97). Project investigators responsible for research oversight of regional hubs will have a key role in ensuring the project meets local ethics committee requirements.

## Discussion

By enhancing the network that has been established through the ABCD Project, the partnership will make a substantial contribution to the evidence base on the design and effective implementation of quality improvement efforts in primary health care, to capacity building, to effective translation of research evidence into policy and service delivery, to strengthening primary health care systems and practice, and to improving Indigenous health outcomes.

## Competing interests

The authors declare that they have no competing interests.

## Authors' contributions

RB and DS played a lead role in conceptualisation of study design, development of measurement tools, and drafting the grant application and this manuscript. CS, JS, KR, DJS, TN, IA, CC, TW, ST, RM, HB, EM, DL, RW and KG contributed to further development of the study design and grant application. RB, CS, JS, KR, DJS, TN, IA, CC, TW, ST, RM, HB, EM, DL, RW, HG and AS played important roles in establishing research and industry partnerships. All authors read and approved the final manuscript.

## Pre-publication history

The pre-publication history for this paper can be accessed here:

http://www.biomedcentral.com/1472-6963/10/129/prepub
